# Germanium Negative Capacitance Field Effect Transistors: Impacts of Zr Composition in Hf_1−*x*_Zr_*x*_O_2_

**DOI:** 10.1186/s11671-019-2927-9

**Published:** 2019-04-04

**Authors:** Yue Peng, Yan Liu, Genquan Han, Jincheng Zhang, Yue Hao

**Affiliations:** State Key Discipline Laboratory of Wide Band Gap Semiconductor Technology, School of Microelectronics, Xidian University, Xi’an, 710071 China

**Keywords:** Ferroelectric, Negative capacitance, Hysteresis, Subthreshold swing, FET

## Abstract

Germanium (Ge) negative capacitance field-effect transistors (NCFETs) with various Zr compositions in Hf_1−*x*_Zr_*x*_O_2_ (*x* = 0.33, 0.48, and 0.67) are fabricated and characterized. For each Zr composition, the NCFET exhibits the sudden drop in some points of subthreshold swing (SS), which is induced by the NC effect. Drive current *I*_DS_ increases with the increase of annealing temperature, which should be due to the reduced source/drain resistance and improved carrier mobility. The steep SS points are repeatable and stable through multiple DC sweeping measurement proving that they are induced by the NC effect. The values of gate voltage *V*_GS_ corresponding to steep SS are consistent and clockwise *I*_DS_-*V*_GS_ are maintained through the multiple DC sweeps. At fixed annealing temperature, NC device with Hf_0.52_Zr_0.48_O_2_ achieves the higher *I*_DS_ but larger hysteresis compared to the other compositions. NCFET with Hf_0.67_Zr_0.33_O_2_ can obtain the excellent performance with hysteresis-free curves and high *I*_DS_.

## Background

The ferroelectric negative capacitance field-effect transistor (NCFET) with a ferroelectric film inserted into gate stack is a promising candidate for the low-power dissipation applications owing to its ability to overcome the fundamental limitation in subthreshold swing (SS) for the conventional metal-oxide-semiconductor field-effect transistor (MOSFET) [1]. The negative capacitance (NC) phenomena in NCFETs have been extensively studied in different channel materials, including silicon (Si) [2, 3], germanium (Ge) [4], germanium-tin (GeSn) [5], III–V [6], and 2D materials [7]. Also, the NC characteristics have been demonstrated in NCFETs with various ferroelectrics, such as BiFeO_3_ [8], PbZrTiO_3_ (PZT) [9], PVDF [10], and Hf_1−*x*_Zr_*x*_O_2_ [11]. Compared to other ferroelectrics, Hf_1−*x*_Zr_x_O_2_ has the advantage of being compatible with CMOS integration. Experimental studies have shown that the electrical performance of NCFETs can be optimized by varying the thickness and area of Hf_1−*x*_Zr_*x*_O_2_, which affects the matching between MOS capacitance (*C*_MOS_) and ferroelectric capacitance (*C*_FE_) [12, 13]. It is expected that the Zr composition in Hf_1−*x*_Zr_*x*_O_2_ also has a great impact on the performance of NCFETs, because it determines the ferroelectric properties of Hf_1−*x*_Zr_*x*_O_2_. However, there is still a lack of a detailed study on the impacts of Zr composition on the electrical characteristics of NCFETs.

In this paper, we comprehensively study the influences of the annealing temperature and the Zr composition on the performance of Ge NCFET.

## Methods

**Fig. 1:**
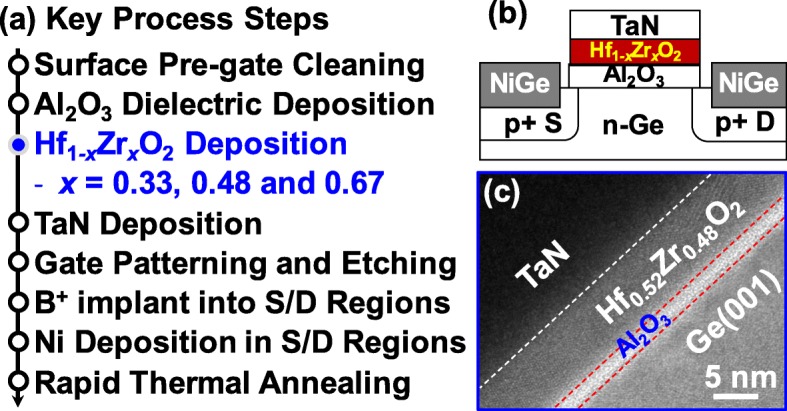
(**a**) Key process steps for the fabrication of Ge NCFETs with the different Zr compositions in Hf_1*−x*_Zr_*x*_O_2_ ferroelectrics. (**b**) Schematic of the fabricated NC transistor. (**c**) TEM image of the gate stack of NC device illustrating the 7 nm H_0.52_Zr_0.48_O_2_ layer and 2 nm Al_2_O_3_ layer

Key process steps for fabricating Ge p-channel NCFETs with the different Zr compositions in Hf_1−*x*_Zr_*x*_O_2_ are shown in Fig. 1(a). After the pregate cleaning, n-Ge (001) substrates were loaded into the atom layer deposition (ALD) chamber. A thin Al_2_O_3_ (25 cycles) film was deposited, which was followed by the O_3_ passivation. Then, the Hf_1-*x*_Zr_*x*_O_2_ films (x = 0.33, 0.48 and 0.67) were deposited in the same ALD chamber using [(CH_3_)_2_N]_4_Hf (TDMAHf), [(CH_3_)_2_N]_4_Zr (TDMAZr) and H_2_O as the Hf, Zr, and O precursors, respectively. After that, the TaN metal gate was deposited using the reactive sputtering. After gate patterning and etching, boron ions (B^+^) were implanted into source/drain (S/D) regions at an energy of 20 keV and a dose of 1 × 10^15^ cm^−2^. Non-self-aligned S/D metals were formed by lift-off process. Finally, rapid thermal annealing (RTA) was carried out at various temperatures for dopant activation, S/D metallization, and crystallization of Hf_1−*x*_Zr_*x*_O_2_ film. Ge control pMOSFETs with the Al_2_O_3_/HfO_2_ stack was also fabricated.

Figure 1(b) shows the schematic of the fabricated NCFET. High-resolution transmission electron microscope (HRTEM) image in Fig. 1(c) shows the gate stack on Ge channel of device with Hf_0.52_Zr_0.48_O_2_ ferroelectric. The thicknesses of Al_2_O_3_ and Hf_0.52_Zr_0.48_O_2_ layers are 2 nm and 7 nm, respectively.

**Fig. 2:**
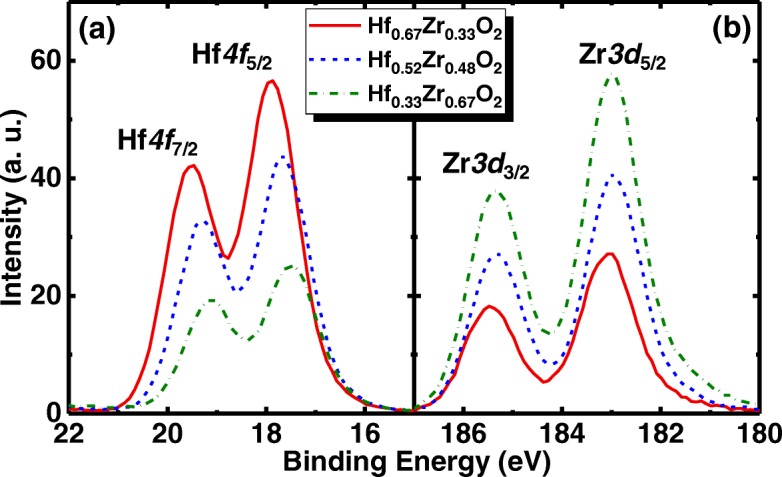
(**a**) Hf *4f* and (**b**) Zr *3d* core level spectra for the Hf_1−*x*_Zr_*x*_O_2_ samples with the different Zr compositions

To confirm the stoichiometries of Hf_1−*x*_Zr_*x*_O_2_, the X-ray photoelectron spectroscopy (XPS) measurement was carried out. Figure 2(a) and (b) show the Hf*4f* and Zr*3d* photoelectron core level spectra, respectively, for the Hf_0.67_Zr_0.33_O_2_, Hf_0.52_Zr_0.48_O_2_, and Hf_0.33_Zr_0.67_O_2_ films. The material compositions were calculated based on the area ratio of the peaks and the corresponding sensitivity factors. The two peaks of Zr*3d*_5/2_ and Zr*3d*_3/2_ have a spin-orbital splitting of 2.4 eV, which is consisted with Refs. [14, 15]. With the increment of Zr composition in Hf_1−*x*_Zr_*x*_O_2_, Zr*3d*, and Hf*4f* peaks shift to the lower energy direction.

**Fig. 3:**
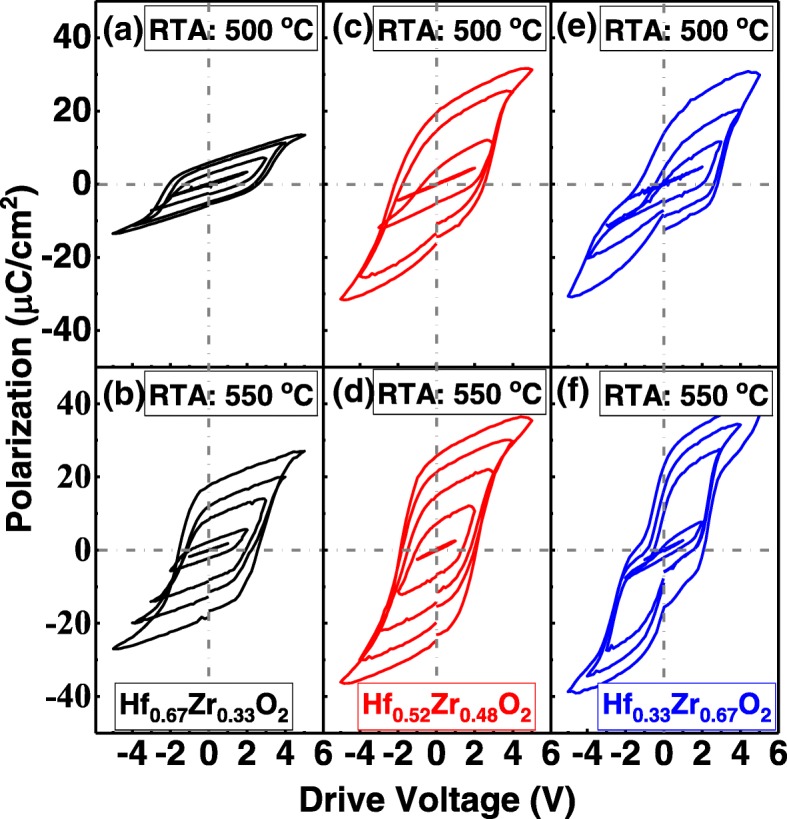
Measured P-V curves of the Hf_1-x_ZrxO2 films with different Zr compositions annealed at 500 and 550 ^o^C. (**a**) and (**b**) are the Hf_0.67_Zr_0.33_O_2_ film annealed at 500 and 550 ^o^C, respectively. (**c**) and (**d**) are the Hf_0.52_Zr_0.48_O_2_ film annealed at 500 and 550 ^o^C, respectively. (**e**) and (**f**) are the Hf_0.33_Zr_0.67_O_2_ film annealed at 500 and 550 ^o^C, respectively. With the post annealing temperature increases from 500 to 550 ^o^C, the P-V curves of the Hf_1-x_Zr_x_O_2_ tend to be saturated in a sub-loop state. An evolution ferroelectric to an antiferroelectric-like behavior is observed with the Zr composition increased

The ferroelectric properties of the Hf_1−*x*_Zr_x_O_2_ films (*x* = 0.33, 0.48, and 0.66) were characterized by the polarization *P* vs. drive voltage *V* hysteresis loops measurement. *P*-*V* loops were recorded on the pristine devices. Figure 3 shows the curves of *P* vs. *V* for TaN/Hf_1−*x*_Zr_*x*_O_2_(10 nm)/TaN samples in a series of drive voltages. With the post-annealing temperature increases from 500 to 550 °C, the *P*-*V* curves of the Hf_1−*x*_Zr_*x*_O_2_ tend to be saturated in a sub-loop state. As the Zr composition increases, the remnant polarization of the film is obviously improved, and the thinning of the hysteresis loop at zero bias is observed, which can be phenomenologically best described as superimposed antiferroelectric-like characteristics [16, 17].

## Results and Discussion

**Fig. 4:**
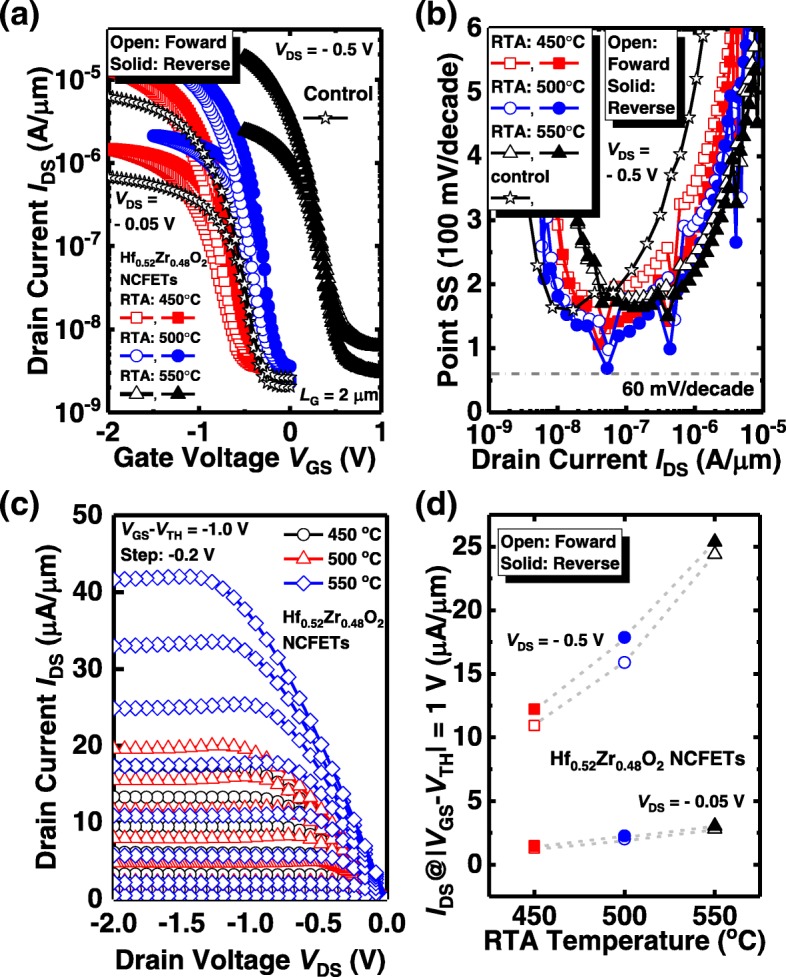
(**a**) Measured *I*_DS_-*V*_GS_ curves for NCFETs with Hf_0.52_Zr_0.48_O_2_ ferroelectric and control device. (**b**) Point SS vs. *I*_DS_ curves showing that NCFETs have the steeper SS compared to control MOSFET. (**c**) *I*_DS_-*V*_DS_ curves for the NCFETs demonstrating the typical NDR phenomena. (**d**) Comparison of the *I*_DS_ for the NCFETs annealed at various temperatures at a gate overdrive of 1 V

Figure 4(a) shows the measured transfer characteristics of Ge NCFETs with Hf_0.52_Zr_0.48_O_2_ ferroelectrics with different annealing temperatures and control device with Al_2_O_3_/HfO_2_ stack dielectric. The control device was annealed at 500 °C. All the devices have a gate length *L*_G_ of 2 μm. The forward and reverse sweeping are indicated by the open and solid symbols, respectively. The NCFETs have a much higher drive current compared to the control device. It is seen that, with the annealing temperature increasing from 450 to 550 °C, the threshold voltage *V*_TH_ of the NC devices shift to the positive *V*_GS_ direction. The NCFETs exhibit a small hysteresis, which becomes negligible with the increasing of RTA temperature. The trapping effect also leads to the hysteresis, but that produces the counterclockwise *I*_DS_-*V*_GS_ loop, opposite to the results induced by ferroelectric switching [18]. Point SS vs. *I*_DS_ curves in Fig. 4(b) show that the NC transistor exhibits the sudden drop in some points of SS, corresponding to the abrupt change of *I*_DS_ induced by the NC effect [19]. It is observed that NCFETs achieve the improved SS characteristics compared to the control device. We found that the sudden drop points of the devices are consistent at the different annealing temperatures. The measured *I*_DS_-*V*_DS_ curves of the NCFETs with Hf_0.52_Zr_0.48_O_2_ ferroelectric annealed at different temperatures are shown in Fig. 4(c). *I*_DS_-*V*_DS_ curves of the NC transistor show the obvious NDR phenomenon, which is a typical characteristic of NC transistors [20–23]. Figure 4(d) is the plots of the *I*_DS_ of the Ge NCFETs with the Hf_0.52_Zr_0.48_O_2_ ferroelectric layer annealed at 450, 500, and 550 °C, respectively, at *V*_DS_ = − 0.05 V and − 0.5 V, and |*V*_GS_ − *V*_TH_| = 1.0 V. Here, the *V*_TH_ is defined as the *V*_GS_ at *I*_DS_ of 10^−7^ A/μm. *I*_DS_ increases with the increasing of RTA temperature, which is due to the reduced source/drain resistance and improved carrier mobility at the higher annealing temperature.

**Fig. 5:**
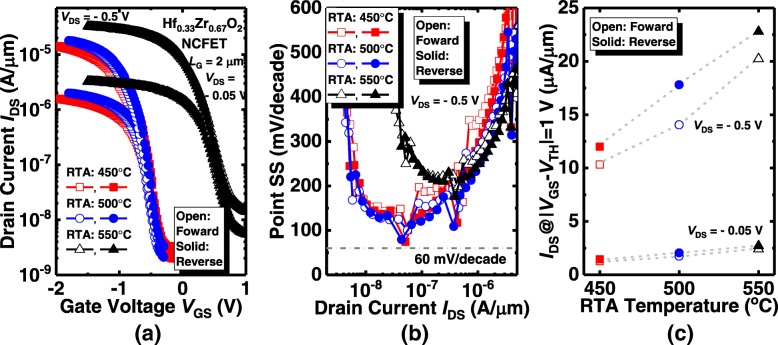
(**a**) Measured transfer characteristics of the Hf_0.33_Zr_0.67_O_2_ NC Ge pFETs annealed from 450 to 550 °C. (**b**) Point SS as a function of *I*_DS_ for the Hf_0.33_Zr_0.67_O_2_ devices. (**c**) *I*_DS_ for the ferroelectric NC transistors with different annealing temperatures at a gate overdrive of 1 V

In addition to the Hf_0.52_Zr_0.48_O_2_ ferroelectric transistor, we also investigate the electrical characteristics of Ge NC transistors with the Hf_0.33_Zr_0.67_O_2_ ferroelectric. Figure 5(a) presents the *I*_DS_-*V*_GS_ characteristics of the devices with Hf_0.33_Zr_0.67_O_2_ with the different annealing temperatures at *V*_DS_ = − 0.05 V and − 0.5 V. Compared to the Hf_0.52_Zr_0.48_O_2_ NC transistors, even smaller hysteresis is obtained. Similar to the Hf_0.52_Zr_0.48_O_2_ NC transistors, as the annealing temperature increases from 450 to 550 °C, *V*_TH_ of the device increase from − 0.63 V to 0.51 V in the forward sweeping at *V*_DS_ = − 0.05 V. Point SS as a function of *I*_DS_ characteristics for the Hf_0.33_Zr_0.67_O_2_ ferroelectric NCFETs are depicted in Fig. 5(b). In addition, devices with 450 °C and 500 °C annealing temperature obtains the more obvious sudden drop in SS in comparison with the 550 °C annealed transistor. The sudden drop points in different annealing temperatures occur at the same gate voltage. Figure 5(c) exhibits forward and reverse *I*_DS_ of the Hf_0.33_Zr_0.67_O_2_ NCFETs at *V*_DS_ = − 0.05 V and − 0.5 V, and |*V*_GS_–*V*_TH_| = 1.0 V. Whether for the forward or reverse sweeping, the *I*_DS_ increases with the annealing temperature, which is consistent with the characteristic of the Hf_0.52_Zr_0.48_O_2_ device.

**Fig. 6:**
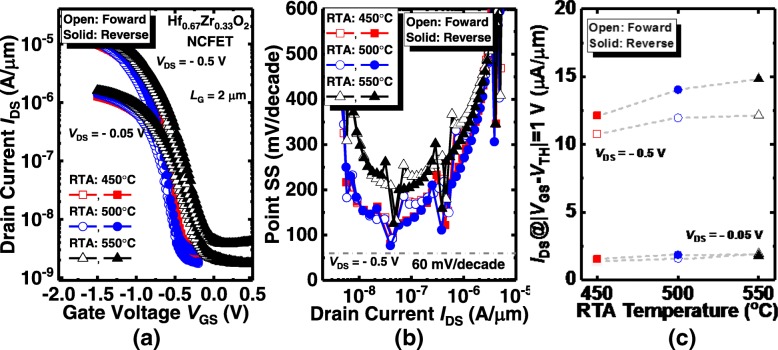
(**a**) Measured *I*_DS_-*V*_GS_ of the Hf_0.67_Zr_0.33_O_2_ NC Ge pFETs annealed at 450 °C, 500 °C, and 550 °C. (**b**) Point SS vs. I_DS_ characteristics of the devices. (**c**) *I*_DS_ for the ferroelectric NC transistors with different annealing temperatures at a gate overdrive of 1 V

We also investigate the electrical performance of Ge NCFET with the smaller Zr composition. The transfer characteristics of the Hf_0.67_Zr_0.33_O_2_ NCFETs annealed at different annealing temperatures are presented in Fig. 6(a). No hysteresis phenomenon is observed. Compared to Hf_0.33_Zr_0.67_O_2_ and Hf_0.52_Zr_0.48_O_2_ devices, the *V*_TH_ shift induced by varying annealing temperature is less pronounced in Hf_0.67_Zr_0.33_O_2_ NCFETs. Point SS vs. *I*_DS_ curves in Fig. 6(b) show that the Hf_0.67_Zr_0.33_O_2_ NC transistor exhibits the sudden drop in some points of SS of NC transistor at *V*_DS_ = − 0.05 V. Figure 6(c) presents the *I*_DS_ of Hf_0.67_Zr_0.33_O_2_ Ge NCFETs annealed at 450 °C, 500 °C, and 550 °C, at *V*_DS_ = − 0.05 V and − 0.5 V, and |*V*_GS_–*V*_TH_| = 1.0 V. Likewise, *I*_DS_ enhances as the RTA temperature increases.

**Fig. 7:**
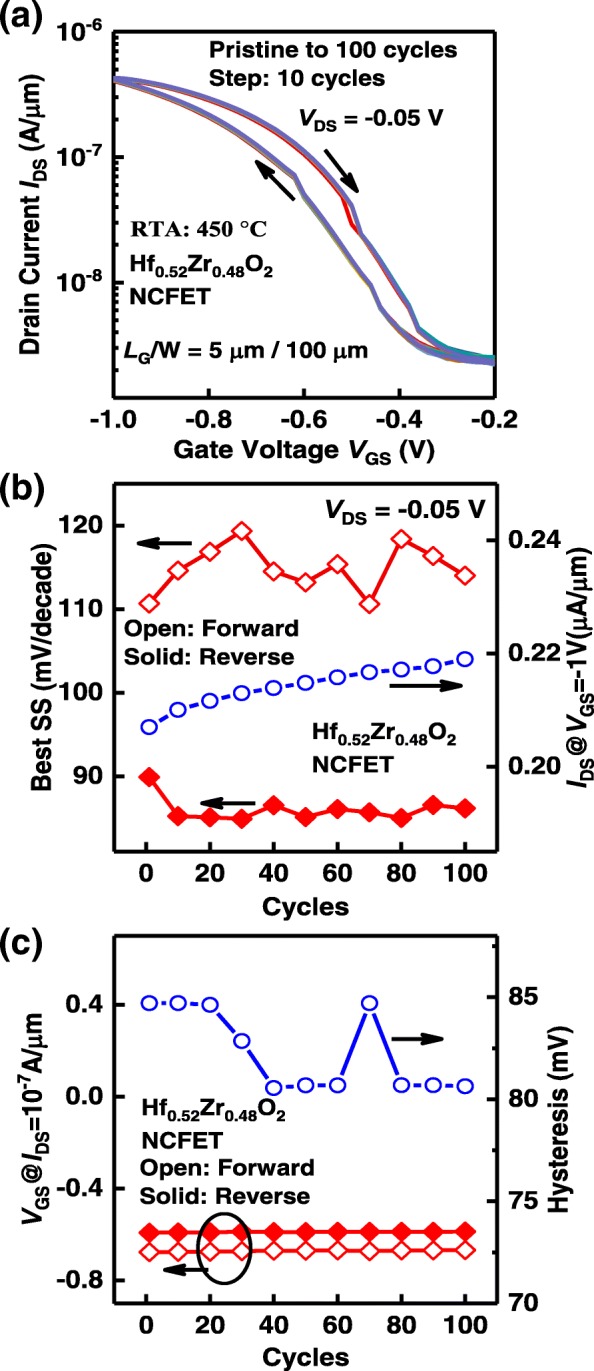
(**a**) Measured *I*_DS_-*V*_GS_ curves of a Hf_0.52_Zr_0.48_O_2_ NC Ge pFET over 100 cycles of DC sweeping. (**b**) Best point SS and *I*_DS_ vs. cycle number. (**c**) Hysteresis characteristics as a function of the number of DC sweeping cycles

The stability of the NC effect induced by the ferroelectric layer of the Hf_0.52_Zr_0.48_O_2_ NCFET was verified by multiple DC sweeping measurements. The measured *I*_DS_-*V*_GS_ curves over 100 cycles of DC sweeping are shown in Fig. 7(a). It can be seen that the values of *V*_GS_ corresponding to steep SS are consistent. In addition, the clockwise *I-V* loops are maintained through the multiple DC sweeps. The steep SS points are repeatable and stable through multiple DC sweeps, which further proves that they are induced by the NC effect. Figure 7(b) presents the best point SS and drive current across the number of sweeping cycles. Figure 7(c) shows the hysteresis characteristics as a function of the number of DC sweeping cycles. Stable *I-V* hysteresis window of ~ 82 mV are seen.

**Fig. 8:**
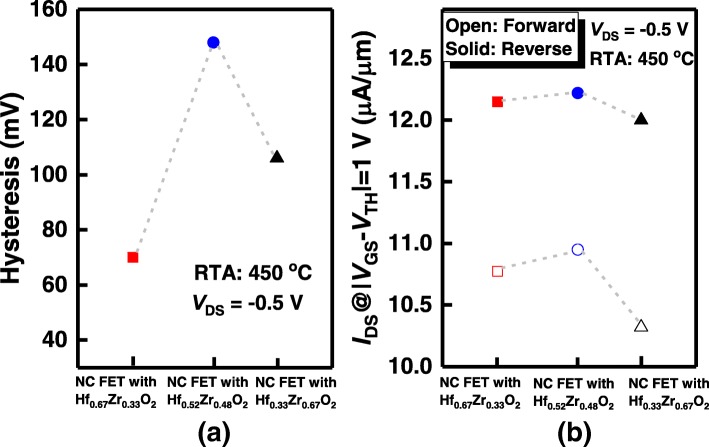
Statistical plots of (**a**) hysteresis and (**b**) *I*_DS_ of Ge NCFET with Hf_1−*x*_Zr_*x*_O_2_ (*x* = 0.33, 0.48, and 0.67)

We summarize the hysteresis and drive current characteristics of Ge NCFETs with different Zr compositions in Hf_1−*x*_Zr_*x*_O_2_ in Fig. 8. As shown in Fig. 8(a), the hysteresis values are 70, 148, and 106 mV for devices with *x* = 0.33, 0.48, and 0.67, respectively, at a *V*_DS_ of – 0.5 V. As the composition increases from 0.33 to 0.48, the hysteresis of the NC device increases significantly. With the further increasing of Zr composition, the hysteresis decreases rapidly. The *I*_DS_ of NCFETs annealed at 450 °C is plotted in Fig. 8(b), at *V*_DS_ = − 0*.*5 V and *V*_GS_ − *V*_TH_ = − 1*.*0 V. Open and solid represent the forward and reverse sweeping, respectively. The NC device with Hf_0.52_Zr_0.48_O_2_ achieves the highest *I*_DS_, but its hysteresis is serious. NCFET with Hf_0.67_Zr_0.33_O_2_ can obtain excellent performance with hysteresis-free curves and high *I*_DS_. As Zr composition increases, the ferroelectric capacitance *C*_fe_ (= 0.3849*P_r_/(*E*_c_*t_fe_) [24]) increases with the increasing of *P*_r_, and meanwhile, the MOS capacitance (*C*_MOS_) rises as well due to the growing permittivity of the HZO film. The *I*_DS_ and hysteresis are determined by |*C*_fe_| and *C*_MOS_ of the transistor. With Zr composition increasing from 0.33 to 0.48, the increase of |*C*_fe_| is speculated to be slower than does the *C*_MOS_, leading to the widening of the hysteresis. Nevertheless, the larger *C*_MOS_ produces a higher *I*_DS_. With the further increase of Zr composition, the increase of |*C*_fe_| is faster than *C*_MOS_, which might provide |*C*_fe_| ≥ *C*_MOS_, reducing the hysteresis of NCFET.

## Conclusions

The impacts of the annealing temperature and Zr composition in Hf_1*−x*_Zr_*x*_O_2_ on the electrical performance of the Ge NCFETs are experimentally studied. The stoichiometries and ferroelectric properties of Hf_1−*x*_Zr_*x*_O_2_ were confirmed by XPS and *P-V* measurements, respectively. NCFETs demonstrate the steep point SS and improved *I*_DS_ compared to the control device, due to the NC effect. The *V*_TH_ and *I*_DS_ of the Hf_1*−x*_Zr_*x*_O_2_ NCFET are greatly affected by the annealing temperature. Multiple DC sweeping measurements show that the stability of the NC effect induced by the ferroelectric layer is achieved in NCFET. Hf_0.67_Zr_0.33_O_2_ NCFET can more easily achieve the hysteresis-free characteristics than the devices with higher Zr composition.

## Abbreviations

Al_2_O_3_: Aluminum oxide
ALD: Atomic layer deposition
BF_2_^+^: Boron fluoride ion
DC: Direct current
Ge: Germanium
GeO_*x*_: Germanium oxide
HF: Hydrofluoric acid
HfO_2_: Hafnium dioxide
HRTEM: High-resolution transmission electron microscope
MOSFETs: Metal-oxide-semiconductor field-effect transistors
NC: Negative capacitance
Ni: Nickel
SS: Subthreshold swing
TaN: Tantalum nitride
TDMAHf: Tetrakis (dimethylamido) hafnium
TDMAZr: Tetrakis (dimethylamido) zirconium
